# A Suggested New Departure in Hospital Administration

**Published:** 1904-11-26

**Authors:** 


### HOSPITAL ADMINISTRATION.

### CONSTRUCTION AND ECONOMICS.

### A SUGGESTED NEW DEPARTURE IN HOSPITAL ADMINISTRATION.

#### I. THE CHAIRMAN-SUPERINTENDENT.—ADVANTAGES.

There has always been a notable difference between the London hospitals and the extra-metropolitan and Scotch hospitals in regard to their committees of management. In London the difficulty has been to procure gentlemen to serve on these committees who, being engaged in active business, were yet sufficiently interested in a particular hospital to give adequate time to the conduct of its affairs. In the provinces and in Scotland the hospital is universally regarded as one of the great institutions of each city or town, where it ministers to the needs of the inhabitants. In consequence a seat on the committee of one of the principal hospitals confers a certain social status and importance, which is highly valued by the most active members of the community. This feeling is honourable to those who possess it, and leads to results of marked importance to the work of the hospitals, as exhibited in their accounts and the general efficiency which characterises their administration. It follows that the chairman of such a hospital holds a post of honour, so that provincial and Scotch hospitals are often able to command the services of the most influential and wealthy citizens. The chairman in such circumstances takes an active part in the administration of the hospital, though he necessarily confines his work to matters affecting the general administration of the charity, whilst he leaves the executive details to be carried out by the paid staff. This system works exceptionally well, especially in the case of large hospitals where the chief executive officer is a medical superintendent, who exercises authority over the whole of the resident staff of the institution, subject only to the committee of management.

Let us contrast this form of administration with another which some think might prove successful in conducting the affairs of a great hospital where there would be no medical superintendent as the chief executive officer, and where the chairman would undertake the duties usually discharged by the principal member of the paid staff, in so far as this is possible for a gentleman who would not reside in the institution, but necessarily has many duties to perform in connection with his own private business elsewhere. Such a chairman-superintendent would not therefore devote many hours daily to the work, although he might be in telephonic touch with the principal officers of the hospital.

A new departure, like this, might lead to some good results, but must also be attended by many disadvantages. Amongst the advantages would probably be the introduction of a number of young, influential, and active men as members of the committee of management. These gentlemen might gather round the chairman-superintendent, being attracted to the work by the activity of the gentleman holding this office, or from feelings of personal friendship to himself. From their number it should be possible to select half-a-dozen men who might each be entrusted with the chairmanship of a sub-committee which could be placed in charge of one department. Thus an estates committee might look after the investment of the funds and the letting of the various properties which the hospital may possess; a dispensary committee might examine the expenditure upon and the cost of drugs, and generally supervise everything connected with the supplies which pass through the hands of the chief dispenser; a stewards' committee might deal with the contracts, and look after the expenditure and control of the provisions and supplies which pass through the hands of the chief steward; a nursing committee might regulate everything which appertains to the department of the matron; a commitee of accounts might supervise the keeping of books, and be in touch with the auditors, so that the general income and expenditure of the hospital might thus be brought frequently under review. In the ordinary course of events this latter committee might be charged with the duty of organising any special appeals which may be issued for money, and everything which has to do with finance. From the business side, assuming that the work was intelligently done, and that each committee has an active chairman of ability and knowledge, the work of a great hospital under such a system should be conducted in a businesslike and thorough manner. We believe that in practice this must at first prove to be the case, but there must always be the danger, that were such a chairman-superintendent to retire, much of the work might suffer, and some at any rate of the departments might fail to be maintained in th highest state of efficiency.

No one can deny that when a hospital has the advantage of a great personality at the head of its administration, providing he is a man of discretion who does not continually mix himself up with the small executive details of the daily work of the institution, the hospital may reap much that is to its advantage. Such a gentleman will take great personal pride in interesting as many people as possible in the work of his hospital. He will spare no pains to preach its efficiency, and to learn which people possess the means, and which are most likely to be influenced by his appeal for funds. From his position, if he is the right man in the right place, he should be able to see personally a large number of rich and influential people who would only otherwise be approachable through the post. It follows, as experience has proved, that such a chairman-superintendent may bring large contributions into the treasury and secure gifts of importance which, without his aid, might never reach the particular hospital at all. All this is undoubtedly to the good, and we would be the first to recognise the importance and self-denial of much of the service which could thus be rendered to the hospital by a chairman-superintendent. It would, however, be very difficult for any man, however able, intelligent and enthusiastic he might be, to gradually assimilate to himself the entire control of a great hospital without creating a sense of dependence, and it may be even of submission, to his particular views and wishes, which in the end might bring about a state of affairs that could not make for the highest efficiency in administration.

Having now stated some of the chief advantages which might accrue to a hospital which was under the direct and individual control of a chairman-superintendent, we propose in a further article to deal with the disadvantages of adopting this new departure in the case of a great hospital.

